# Effects of sling exercises on pain, function, and corticomuscular functional connectivity in individuals with chronic low back pain- preliminary study

**DOI:** 10.1371/journal.pone.0288405

**Published:** 2023-11-30

**Authors:** Bo-Jhen Chen, Tzu-Ying Liu, Hsin-Chi Wu, Mei-Wun Tsai, Shun-Hwa Wei, Li-Wei Chou

**Affiliations:** 1 Department of Rehabilitation Medicine, Taipei Tzu Chi Hospital, Buddhist Tzu Chi Medical Foundation, Hualien, Taiwan (R.O.C.); 2 Department of Physical Therapy and Assistive Technology, National Yang Ming Chao Tung University, Hsinchu, Taiwan (R.O.C.); 3 Department of Medicine, Tzu Chi University, Hualien, Taiwan (R.O.C.); King Khalid University, SAUDI ARABIA

## Abstract

**Background:**

Individuals with chronic low back pain (CLBP) exhibit altered brain function and trunk muscle activation.

**Aim:**

This study examined the effects of sling exercises on pain, function, and corticomuscular coherence (CMC) in healthy adults and individuals with CLBP.

**Methods:**

Eight individuals with CLBP and 15 healthy adults received sling exercise training for 6 weeks. Before and after training, participants performed two motor tasks: rapid arm lifts and repeated trunk flexion–extension tasks, and electromyography of the trunk muscles and electroencephalography of the sensorimotor cortex were recorded. Chi-squared test and Mann–Whitney U tests were used for between group comparison, and Wilcoxon signed-rank tests were used for pre- and post-training comparison. Spearman’s Rank Correlation Coefficient (R_s_) was used to identify for the relationship between motor performance and Corticomuscular coherence.

**Results:**

Sling exercises significantly improved pain (median from 3 to 1, p = .01) and Oswestry Disability Index scores (median from 2.5 to 2, p = .03) in the CLBP group. During rapid arm lifts, individuals with CLBP showed lower beta CMC of the transverse abdominis and internal oblique (Tra/IO) (0.8 vs. 0.49, p = .01) and lumbar erector spinae (0.70 vs. 0.38, p = .04) than the control group at baseline. During trunk flexion–extension, the CLBP group showed higher gamma CMC of the left Tra/IO than the control group at baseline (0.28 vs. 0.16 , p = .001). After training, all CMC became statistically non-significant between groups. The training induced improvement in anticipatory activation of the Tra/IO was positively correlated with the beta CMC (r_s_ = 0.7851, p = .02).

**Conclusion:**

A 6-week sling exercises diminished pain and disability in patients with CLBP and improved the anticipatory activation and CMC in some trunk muscles. These improvements were associated with training induced changes in corticomuscular connectivity in individuals with CLBP.

## Introduction

Individuals with nonspecific chronic low back pain (CLBP) present with delayed anticipatory activation of their deep trunk muscles during activities, e.g. during rapid arm lift task [1] and repeated trunk flexion–extension tasks [2, 3]. This phenomenon is observed during not only trunk movement but also sudden limb movement, presumably to provide trunk stability [1, 4].

Exercises on a sling or an unstable surface could induce higher levels of trunk muscle activation than those performed on a stable surface. A meta-analysis indicated that core stability exercises produced short-term reduced pain and disability than general exercise did for patients with CLBP [5]. Although one systematic review indicated that sling exercises achieved promising outcomes for pain and function among patients with CLBP [6], the reduction of symptoms could not be fully explained through structural or functional changes of the trunk muscles [7]. As perception of chronic pain involves the interplay of various systems, several mechanisms have been postulated, such as neuromuscular or neurophysiological alternations. Nevertheless, how these mechanisms mediate pain reduction after intervention remain unclear [8].

During voluntary movement, synchronized oscillatory activities occur between the primary sensorimotor cortex and the corresponding muscles [9]. The level of synchronized oscillation indicates the strength of functional connectivity between the sensorimotor cortex and active muscles and is commonly quantified as corticomuscular coherence (CMC) [10]. Studies have demonstrated that during voluntary movement, synchronized oscillation between the motor cortex and active muscles is dominant in the beta (13–30 Hz) and gamma (30–60 Hz) frequency bands [10, 11]. Beta CMC is related to isometric and low to moderate contractions, whereas gamma CMC is the most dominant during dynamic movement and high levels of muscle contractions [12]. The level of CMC reveals the physiological status of the neuromuscular system. Previous literatures have validated that aging [13, 14] and neurological disorders [15–18] decreased CMC. Exercise and training would enhance the CMC in healthy adults as well as athletes [19, 20].

Despite the fact that CLBP induces reorganization in the motor cortex, resulting in deficits in descending drive and decreased trunk control [21, 22], how CLBP affects functional connectivity between the motor cortex and trunk muscles remain unclear. Furthermore, whether training-related symptom reduction attributes to enhanced CMC has not been explored. Therefore, this study investigated the effects of sling exercises on pain, function, and CMC in patients with CLBP.

## Materials and methods

### Participants

Patients who had been diagnosed with CLBP for more than 3 months and who were 20–50 years old were recruited for this study. Patients who presented spinal stenosis/fracture, radiculopathy, or history of surgery were excluded. Healthy adults aged 20–50 years without neurological diseases were included in the control group. Subjects were recruited from October of 2014 to October of 2015. Their demographic data were roughly matched to the CLBP group when enrolled into the study.

### Ethics statement

This study was approved by the Institutional Review Board of National Yang Ming Chao Tung University (YM103090E), and Taipei Tzu Chi Hospital, Buddhist Tzu Chi Medical Foundation (03-XD14-039), respectively. Written informed consent was obtained from each participant prior to assessment and training.

### Training

Both the healthy control and CLBP groups performed 40 minutes of sling exercises twice a week for 6 weeks. Previous studies have applied similar designs in which the sling exercise programs ensured high activation of the trunk muscles, e.g. chess press, hamstring curl, hip abduction in plank, and single leg squat [6]. Participants practiced the exercises with a TRX Home Sling Training Kit (Fitness Anywhere LLC, USA) under the supervision of a licensed physical therapist. Exercise intensity and rest were adjusted according to each participant’s performance to prevent fatigue or compensatory patterns.

### Outcome measures

Pain, disability, and neuromuscular activities during functional tasks were recorded and analyzed. The Numeric Pain Rating Scale was used to assess the severity of pain, where 0 indicated no pain and 10 indicated maximal imaginable pain. The Oswestry Disability Index (ODI) was used to evaluate the level of disability. The ODI questionnaire comprised 10 dimensions of daily function, and a higher score on a 0–5-point scale indicated more severe disability. The Chinese version of ODI has been well validated with high test–retest reliability for assessing disability in patients with low back pain [23].

To evaluate neuromuscular activity during two functional tasks, we measured the anticipatory activation of the transverse abdominis and internal oblique (Tra/IO) with reference to the anterior deltoid during rapid arm lifts. The activity between the brain and trunk muscles, during a repetitive trunk flexion–extension task, was simultaneously recorded and synchronized to calculate corticomuscular (electroencephalogram [EEG]–electromyography [EMG]) coherence. This approach has been validated by transcranial magnetic stimulation induced motor evoked potentials, in which the corticomuscular coupling occurred between the M1 and effector muscles [24].

### Testing procedures

A pair of silver/silver chloride electrodes (Foam Electrodes, Cardinal Health, USA) were securely attached to the participants’ skin above the targeted muscles with an interelectrode distance of 1.5 cm. Skin was first cleaned with alcohol pads to reduce impedance. The placement of each electrode was determined according to previous studies. The right anterior deltoid’s electrode was placed on the belly [25], the left Tra/IO electrodes were placed 2 cm medially and anteriorly to the left anterior superior iliac spine, and left thoracic erector spinae (TES) and lumbar erector spinae (LES) were placed 5 cm lateral to the T9 and L3 vertebrae respectively [26]. Three 5-second maximal voluntary isometric contractions of each muscle were recorded and averaged for subsequent EMG amplitude normalization. The EEG signals of the bilateral primary motor and somatosensory cortices were collected with a 16-channel EEG system (actiCAP with QuickAmp, Brain Products GmbH, Germany).

For the anticipatory activation test, participants were requested to stand comfortably with their feet shoulder-width apart and hands resting on the side of each leg. They then lifted the resting right arm to the shoulder level as quickly as possible for 10 consecutive times [1]. The timing of muscle activation of the Tra/IO related to the deltoid was recorded. At the meantime, the muscle activities of Tra/IO and LES were synchronized with the EEG signals.

For the repetitive trunk flexion–extension task, participants held a 5-kg object, bent forward to knee height, and returned to their initial standing position [2]. This movement, which lasted 3 seconds, was also repeated 10 times. A practice session followed by 5 minutes of rest was permitted to allow patients to familiarize themselves with the procedure before commencing the formal tests. A metronome was used to standardize the speed. The muscle activities of Tra/IO, TES and LES were synchronized with the EEG signals.

### Data processing

The surface electromyography of the right anterior deltoid, left TES, LES, and Tra/IO was recorded using a wireless EMG system (MP150WMW, BIOPAC System. Inc, USA). Raw EMG signals were sampled (1000 Hz), amplified (2000 times) and bandpass filtered (3–450 Hz) (AcqKnowledge software, MP150WMW, BIOPAC System. Inc, USA). The common mode rejection ratio was 110 dB at 50/60 Hz. The time points of the muscle activation of the anterior deltoid and Tra/IO during the rapid arm lifts were identified to examine the anticipatory activation of the Tra/IO. The EMG signal of the Tra/IO during resting (baseline) and rapid arm lifts was root-mean-squared with 30-ms moving windows. The averaged EMG amplitude plus 3 standard deviations during baseline was set as the threshold of the defined muscle activation. The timing of the activation of each muscle was determined by the point at which the EMG amplitude exceeded the activation threshold. The time differences between the activation of the anterior deltoid and Tra/IO were then calculated. Negative values indicated the anticipatory activation of the Tra/IO occurred prior that of the deltoid [1].

The EEG signals were sampled (1000 Hz) and filtered (0.5–70 Hz), and then synchronized with the EMG signals for corticomuscular coherence analysis. To focus on the cortex region that corresponded to the trunk, we selected EEG channels Cz, C1, and C2 for coherence analysis [27]. EEG and EMG signals were processed offline with MATLAB (The MathWorks, USA) to calculate the coherence according to the following equation [28, 29]:

$$|{\text{c}}_{\text{xy}}\text{(f)}|\text{=}\frac{{\left|\bar{{\text{P}}_{\text{xy}}\text{(f)}}\right|}^{2}}{\bar{{\text{P}}_{\text{xx}}\text{(f)}}\cdot \bar{{\text{P}}_{\text{yy}}\text{(f)}}}$$ (1)

where Cxy(f) denotes the linear coherence between EEG and EMG at frequency (f). Pxx(f) and Pyy(f) represent the power spectral density (PSD) of either EEG or EMG in the frequency domains, and Pxy(f) represents the cross PSD between EEG and EMG. Signals within a 2048-sample epoch with 50% overlap were analyzed. Each signal length was 30 s with 0.98-Hz frequency resolution [30].

Because only the coherence values surpassed the critical threshold are considered physiologically meaningful functional connections [27], we calculated critical threshold of coherence (CT) with a significance level of 0.05 (α) by using Eq 2 [28, 31].


$$\text{CT}=1-{\left(1-\alpha \right)}^{\frac{1}{\left(\hat{\text{k}}-1\right)}}$$ (2)

In Eq (2), $\tilde{k}$ represents the number of overlapped epochs and was calculated with the following Eq (3), where k represents the number of epochs without overlapping.


$$\tilde{k}=\frac{k}{C_{w}(D)}$$ (3)

To calculate *C*_*w*_(*D*), the following two equations (Eqs 4 and 5) were used:

$${\text{C}}_{w}(D)=1+2\left(\frac{k-1}{k}\right)\rho_{w}^{2}(D)$$ (4)

$$\rho_{w}(D)=\frac{\sum_{t=0}^{L-D-1}w_{L}(t)w_{L}(t+D)}{\sum_{t=0}^{L-1}w_{L}^{2}(t)}$$ (5)

where D represents the length that two signals were not overlapped and L represents the length of the epoch.

We focused on beta (15–30 Hz) and low gamma (30–60 Hz) band coherences [12]. The summation of the area that exceeded the critical threshold within each frequency band was calculated, and the largest coherence value among Cz, C1, and C2 was used for later comparison.

### Statistical analysis

Non-parametric statistics were performed to compare the median between and within groups before and after training. Chi-squared test or Mann–Whitney U test was used to compare demographic characteristics between the two groups, and Wilcoxon signed-rank test was used to compare the outcomes pre- and post-training. These values were presented as the median and the first and third interquartile range (Q1—Q3). Spearman correlation was performed to explore the relationship between the change scores and between each outcome measure. All analyses were performed with SPSS Statistics 21.0 (SPSS Inc, USA), and the significance level was set at p value < 0.05 with two-tailed tests.

## Results

### Participants’ characteristics

**Fig 1:**
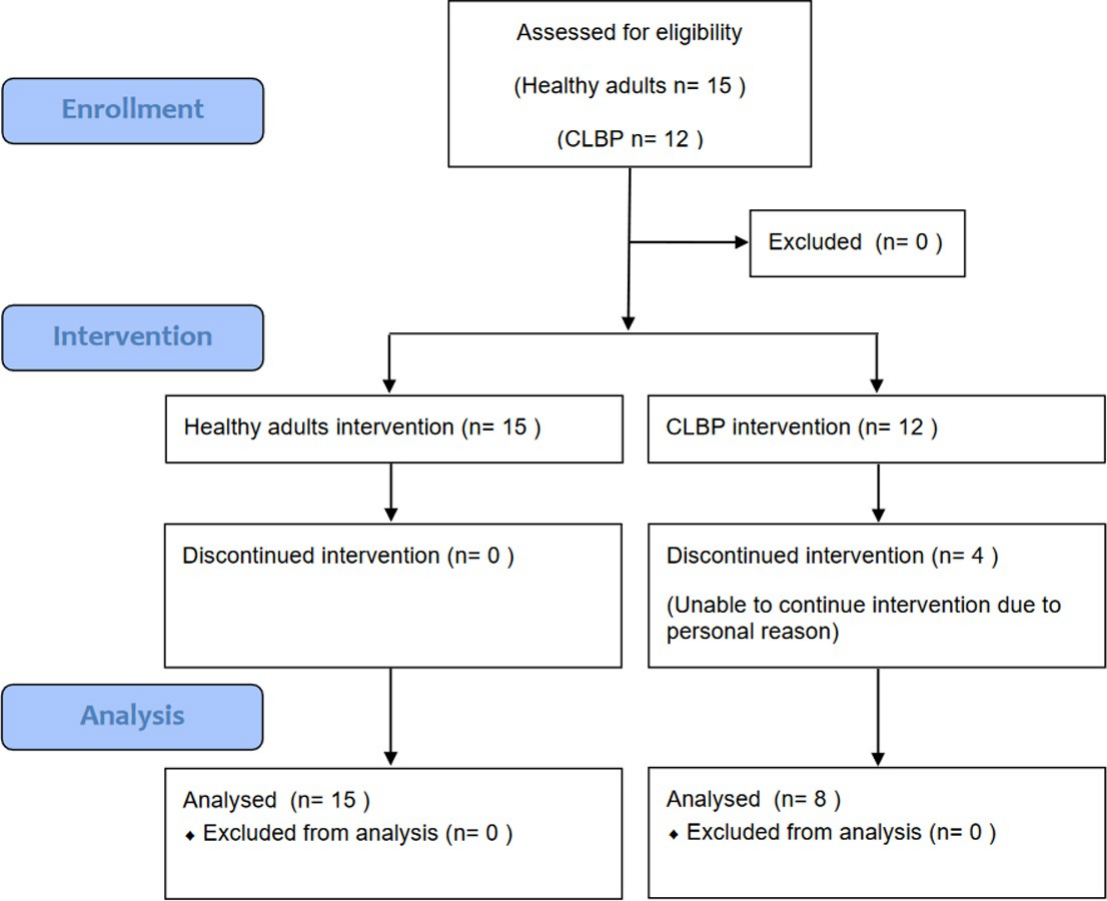
Consort flow chart.

Eight patients, three of whom were females (37.5%), with CLBP were recruited for this study. They were all right-handed and with a relatively sedentary lifestyle. Fifteen healthy individuals, nine of whom were females (60%), were enrolled as the healthy controls (Fig 1). Their demographic data were not significantly different at baseline between groups except absence of low back pain (Table 1). All participants completed the training program and the outcome assessment. No incidence of increased pain or adverse event was reported by completion of the training program.

**Table 1:** Demographic data of the two groups.

	Patient No.	Age (yr)	Sex	Height (cm)	Weight (kg)	Symptoms		Pain	ODI
						Duration (yr)	Side		
**Patient**	1	35	M	170	71	2	Left	2	7
	2	49	M	170	56	8	Right	3	5
	3	22	F	157	50	4	middle	3	2
	4	23	F	167	58	8	Left	5	2
	5	23	F	163	60	5	both	4	6
	6	21	M	174	68	3	Left	5	3
	7	20	M	183	78	0.25	middle	2	2
	8	24	M	168	53	4	middle	3	2
**Median (Q1—Q3)**	**Patient (n = 8)**	23 (21.75–26.75)		169 (166–171)	59 (55.25–68.75)	4 (2.75–5.75)		3 (2.75–4.25)	2.5 (2–5.25)
	**Healthy (n = 15)**	23 (21–24.5)		167 (162.5–171.5)	59 (53–67)	N/A	N/A	N/A	N/A
**P value**		0.37	0.3	0.81	0.72				

yr: year; M: male; F: female; ODI: Oswestry Disability Index.

### Effectiveness on pain and disability

Pain and disability scores were only measured for the CLBP group. After receiving sling exercise training, a significant decrease was noted in pain scores (3 (2.75–4.25) to 1 (1–2), p = .01). Additionally, the disability level according to the ODI significantly improved from 2.5 (2–5.25) to 2 (0.75–2.25)(p = .03).

### Change in anticipatory activation

**Fig 2:**
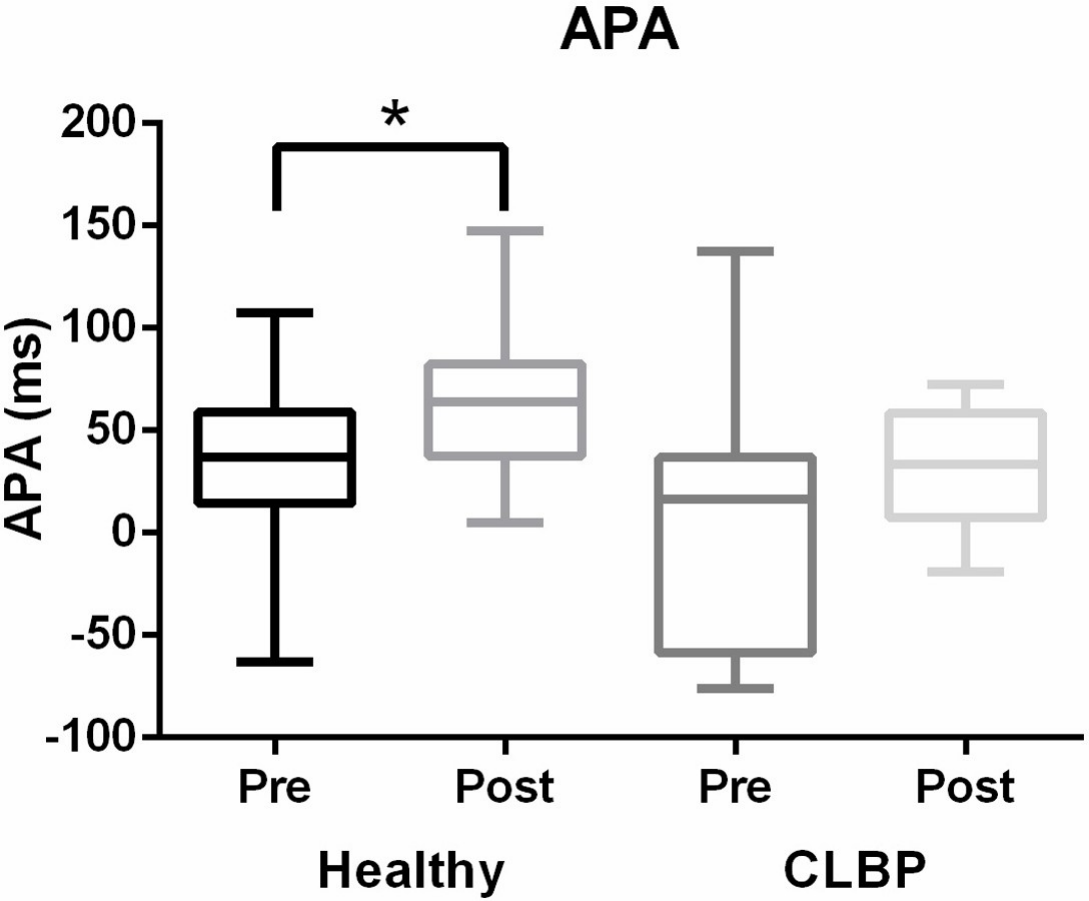
Anticipatory activation for healthy controls and patients with CLBP before (dark) and after (light) sling exercise training. *indicates statistical significance (p < 0.05).

We investigated the activation timing of the left Tra/IO during the rapid arm lift task (Fig 2). Before training, the left Tra/IO contracted 37 (18–51) ms before the right arm movement commencement in healthy controls. After sling exercise training, the anticipatory activation significantly improved, and the left Tra and IO contracted 63 (48–80) ms before arm movement commencement (p = .02). For the CLBP group, Before training, the left Tra/IO was activated 16 (-44–32) ms before arm movement commencement, and after sling exercise training, the anticipatory activation of the left Tra/IO tended to occur earlier, at 33 (14–48) ms before arm movement commencement.

### CMC during rapid arm lifts

**Fig 3:**
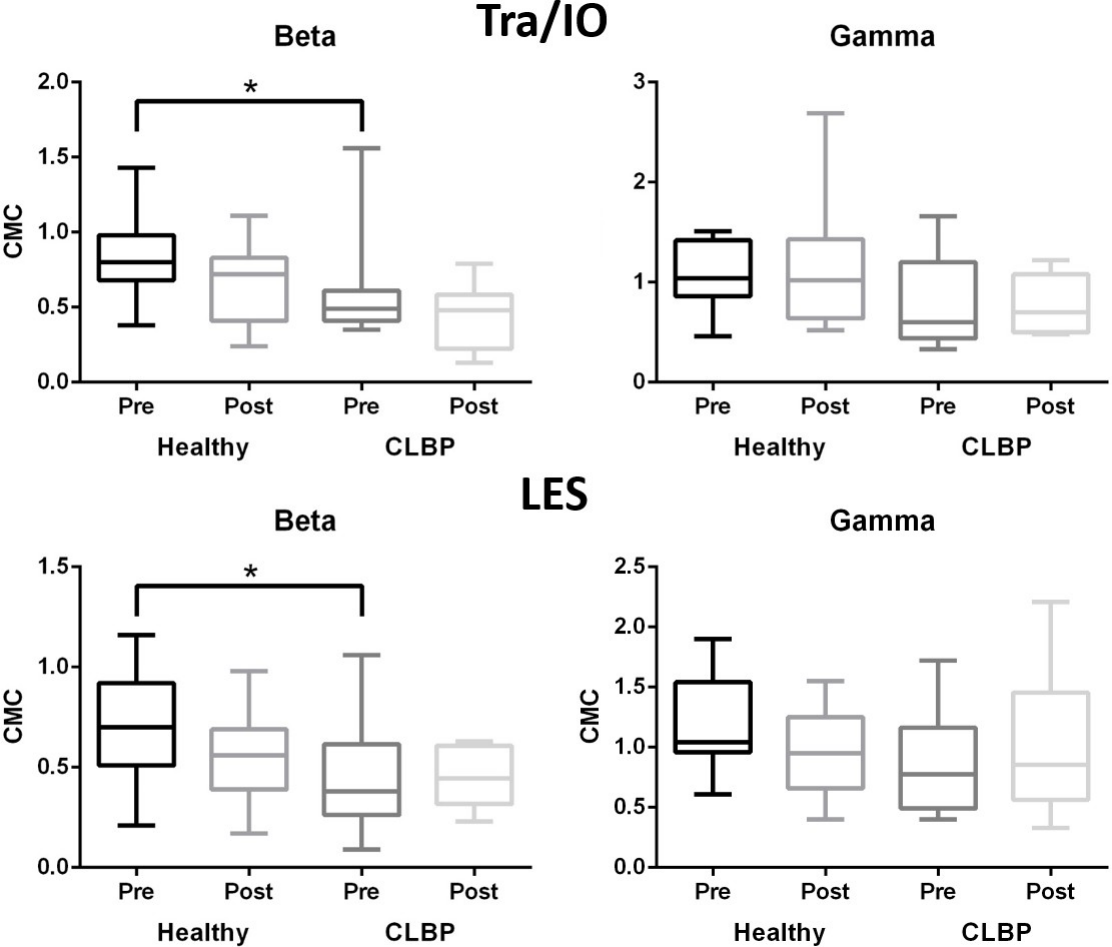
CMCs during rapid arm lift for transverse abdominis (Tra), internal oblique (IO), and lumbar erector spinae (LES) in beta (left) and gamma (right) bands for healthy controls and patients with CLBP before (dark) and after (light) sling exercise training *indicates statistical significance (p < 0.05).

The beta CMC of the left Tra/IO was significantly higher in healthy controls than in individuals with CLBP at baseline (0.8 (0.72–0.97) vs. 0.49 (0.46–0.60), p = .01). However, no statistical difference was observed between the groups after training. Similarly, no statistical difference was observed in the gamma CMC of the left Tra/IO (Fig 3).

For the left LES, the beta CMC was higher in healthy controls than in individuals with CLBP at baseline (0.70 (0.50–0.90) vs. 0.38 (0.26–0.48), p = .04), but not after training (0.56 (0.44–0.66) vs. 0.44 (0.33–0.56), p = .20). No statistical difference was observed in the gamma band (Fig 3).

### CMC during repeated trunk bending

**Fig 4:**
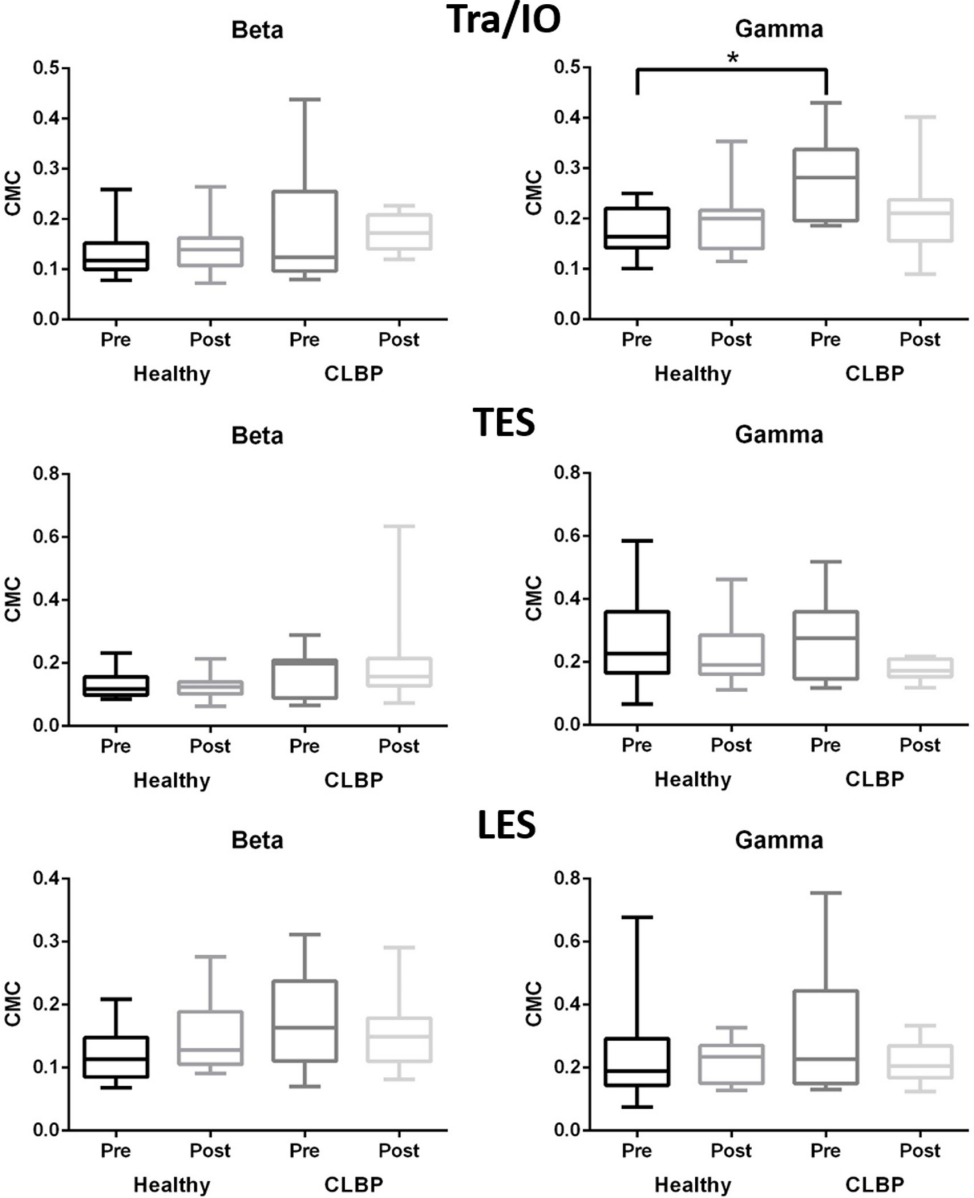
CMCs during repeated trunk bending for Tra/IO, LES, and thoracic erector spinae (TES) in beta (left) and gamma (right) bands for healthy controls and patients with CLBP before (dark) and after (light) sling exercise training *indicates statistical significance (p < 0.05).

During the repeated trunk bending task, no statistical difference was observed in the beta CMC. The gamma CMC of the left Tra/IO was higher in the CLBP group than in the control group at baseline (0.28 (0.21–0.33) vs. 0.16 (0.14–0.21), p = .001). Although not achieving statistical significance, compared with healthy adults, the gamma band of the trunk muscles showed substantial decrease after sling exercise training. Our results showed that the gamma CMC of the Tra/IO, LES, and TES decreased 24%, 36%, and 29%, respectively (Fig 4).

### Association between CMC and other outcome measures

**Fig 5:**
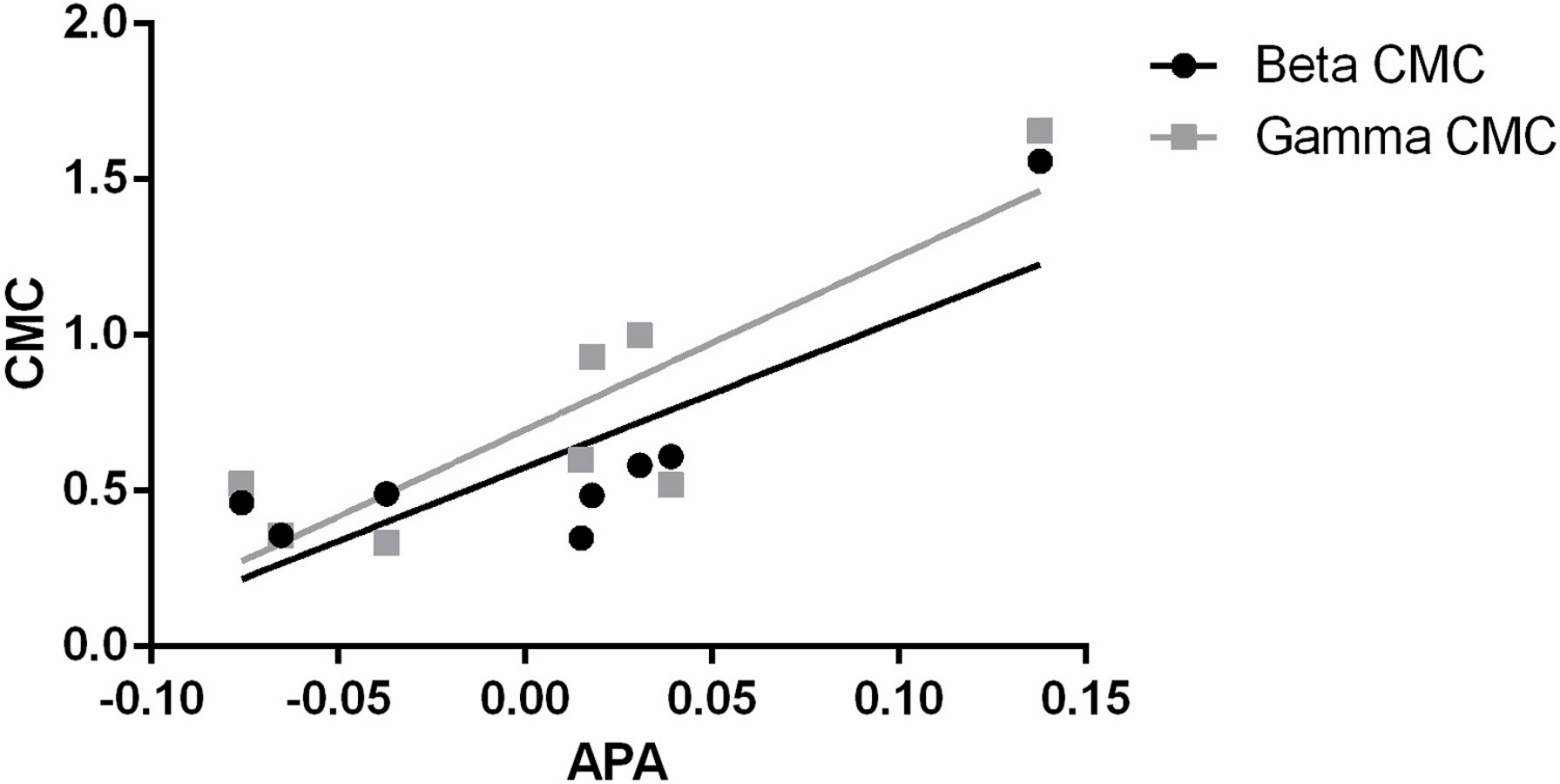
Correlations between anticipatory activation of Tra/IO and CMC in beta (left) and gamma (right) bands.

We observed a high correlation between anticipatory activation and corticomuscular functional connectivity during the rapid arm lift task (Fig 5). The Spearman correlation analysis showed anticipatory activation of the Tra/IO was positively correlated with the beta CMC (r_s_ = 0.7851, p = .02).

**Fig 6:**
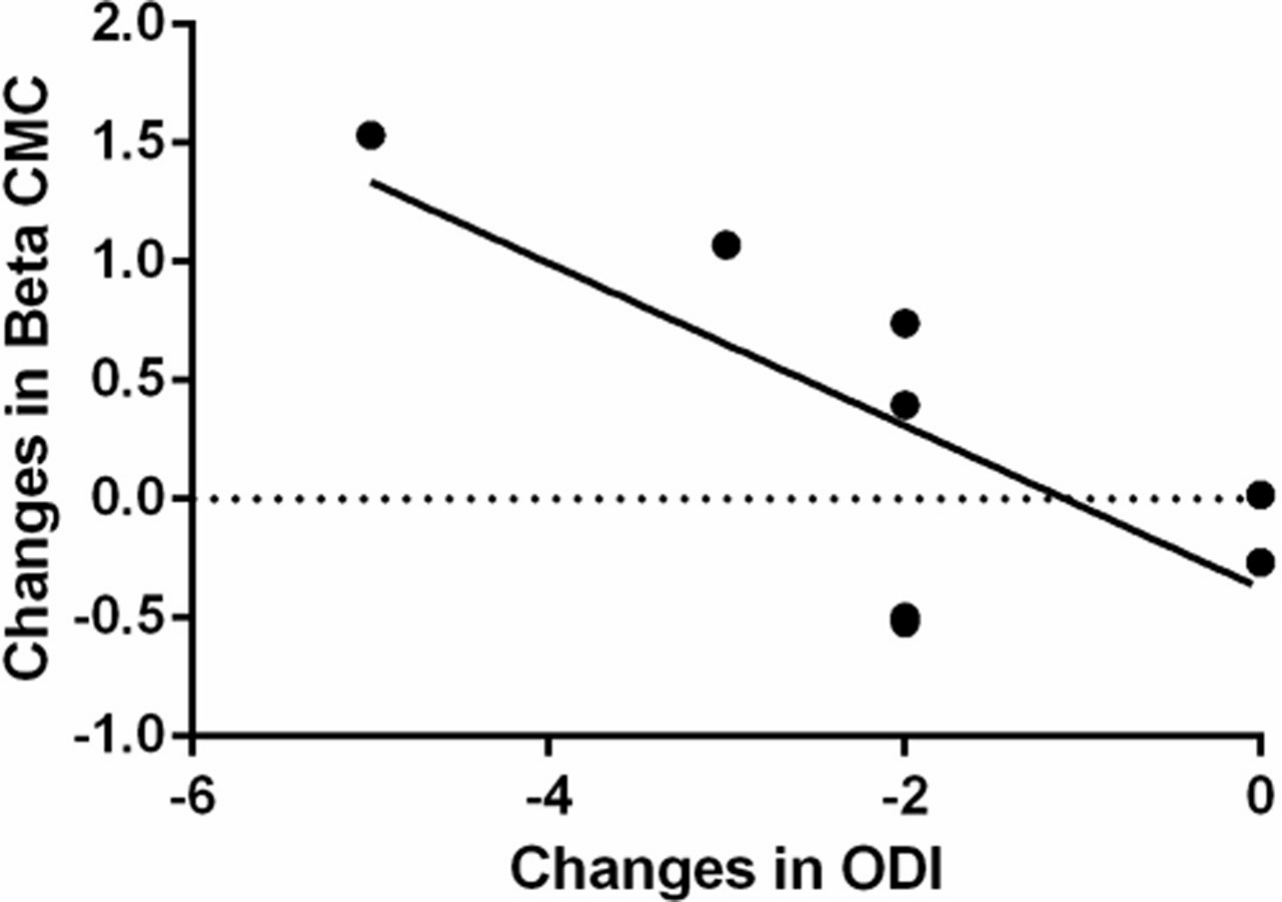
A trend of correlation between sling exercise training–induced increment in the beta CMC of Tra/IO during the repetitive trunk flexion–extension movement and the changes in the Oswestry Disability Index (ODI).

We also observed a trend of correlation (Spearman’s Rank Correlation Coefficient (R_s_)) between training induced functional improvement and CMC. The changes in ODI scores after sling exercise training is negatively correlated with the changes of beta CMC of the Tra/IO during the repetitive trunk flexion–extension movement (r_s_ = 0.6257, p = .09; Fig 6). These results suggested a likelihood of greater functional improvement (ODI) is associated with stronger corticomuscular functional connectivity in the beta band.

## Discussion

We investigated the effects of sling exercise training on pain, function, and trunk muscle control in healthy individuals and patients with CLBP. Sling exercises significantly improved pain and function in individuals with CLBP. Compared with healthy adults, corticomuscular functional connectivity of Tra/IO during rapid arm lift was lower in individuals with CLBP, and higher functional connectivity is associated with better anticipatory activation of Tra/IO. Lastly, training induced improvement in function was associated with the increase in corticomuscular functional connectivity.

### Pain and disability

Our results demonstrated that 6 weeks of sling exercises reduced pain and disability in patients with CLBP. Pain decreased by approximately 2 points on a 10-point scale, which exceeded the minimum clinically significant change [32]. The 6-week sling exercise training also resulted in 55% improvement in ODI scores, which also exceeded the minimum clinically significant change [32]. Systematic reviews have also reported that compared with minimal interventions, motor control exercises were effective for short-term reductions of pain and disability in patients with CLBP [33]. However, functional improvements in the trials that were reported in the systemic review were small and not clinically meaningful. In the present study, reductions of both pain and disability achieved the minimum clinically notable changes. This can be attributed to the characteristics of patients with CLBP in this study, who were relatively young, were committed to a long-term training program, had high expectations of returning to work, and had low pain and disability levels. These characteristics have been suggested as key factors that contribute to greater recovery [34, 35].

### Anticipatory contraction

During the rapid arm lift task, we observed that patients with CLBP exhibited more delayed anticipatory activation of the left Tra and IO than healthy controls did, which is consistent with the findings of previous studies [36, 37]. Our results also indicated that sling exercise training significantly improved healthy controls’ anticipatory activation of the Tra and IO. After sling exercise training, anticipatory activation of the Tra and IO improved from 8 ms to 33 ms in patients with CLBP, which was within the range of healthy adults. However, studies on the effects of core muscle training on anticipatory abdominal activation have reported conflicting results. Brooks et al. [38] compared the effect of trunk-specific or general exercises on anticipatory core muscle activation in patients with CLBP, and they reported improved onset of muscle activation in both exercise groups. Hwang et al. [39] also reported that compared with modality therapy and healthy controls, trunk control training improved the Tra and IO contraction timing. However, studies [40, 41] have also reported modest or no changes among patients with CLBP who performed low-load stability exercises, high-load sling exercises, or general exercises. By comparing the exercise training methods in these studies, we determined that the training amount might play a role in determining the effectiveness of muscle training for anticipatory activation. In studies (including this one) that noted significant improvements in abdominal muscle anticipatory activation, the training programs consisted of more than one session per week. Studies that indicated no significant benefit of training on anticipatory activation, however, implemented only one session per week. Therefore, we recommend a training program consisting of at least two sessions per week for patients with CLBP to achieve significant improvements in abdominal muscle anticipatory activation.

The beta and gamma bands of the left Tra/IO and the beta band of the left LES were higher in healthy controls than in patients with CLBP, which suggests a weakened connection and higher motor threshold of the trunk muscles in individuals with back pain. Furthermore, our correlation analysis demonstrated that patients with CLBP presented a high correlation between the beta CMC and anticipatory activation of the transverse abdominis and internal oblique muscles (r_s_ = 0.78), suggesting that functional connectivity between the sensorimotor cortex and muscles play a vital role in achieving anticipatory activation of the trunk muscles.

### Trunk movement task

With regard to the repeated trunk flexion-extension movement, we noticed a higher coherence of beta and gamma bands in the CLBP group than in the control group before training. Higher activation of the erector spinae during standing was observed in patients with CLBP [42]. Higher activation of the trunk muscles in individuals with CLBP could be due to an impaired flexion–relaxation phenomenon [3], in which the activity of the ES normally decreases toward the end of trunk flexion in healthy adults, but high activation level is maintained throughout the entire trunk movement period in individuals with CLBP [3, 43]. We postulate that over-activation of the trunk muscles in individuals with CLBP resulted in higher CMC during the trunk flexion–extension movement.

Although higher CMC generally indicates greater corticomuscular connectivity and neuromuscular control, this correlation may be invalid if CMC is observed during the motor learning process. In the current study, the gamma band link of the TES significantly decreased in the CLBP group after training. We postulate that the levels of difficulty of and the necessary cortical resources for the motor task determine the level of CMC. When performing a difficult task, the CMC level is likely high. When individuals have mastered the motor task, however, the necessity of allocating cortical resources to execute the given motor task may be low. In this study, patients with CLBP developed greater control of their trunk muscles after sling exercise training, which likely reduced their fear of movement and muscle guarding [44]. Therefore, when patients performed the lifting motor task, the CMC was lower than it was before training. Our previous study that monitored stroke patients’ CMC of their affected hand before, during, and after an 8-week motor training program revealed a similar pattern [45]. In the fourth week, CMC was significantly increased, but hand motor function remained unchanged. In the eighth week, patients’ hand motor function significantly improved, and the CMC of the paretic hand muscle significantly decreased. Similar observations were reported after unipedal postural training [46] and were noted between trained and untrained athletes [20].

### Analysis of corticomuscular coherence

Varied methods have been developed to estimate CMC [47, 48]. Some authors emphasized the peak(s) of the highest coherence during the movement. Of which, majority of the studies reported highest coherence in some specific frequency(ies) during the selected contraction types during specific tasks. However, the functional meaning of CMC related the targeted tasks was unclear. Instead, we calculated the area under the curve and above the confidence level during the two standardized tasks. We postulated that this approach would represent the performance of entire task as a whole spectrum. Nevertheless, this procedure still requires further validation. In addition, we also found moderate negative relationship between the beta CMC and disability scale. The beta CMC and TrA/IO and erector spinae also exhibited moderate to strong positive relationship during rapid arm lift as well as repetitive trunk flexion/extension. In perspective, improvement of symptoms would result from changes of the corticomuscular control.

This study has several limitations. First, we had a small sample size for both healthy controls and patients with CLBP. Second, because patients with CLBP group mild pain and disability, the effects of sling exercises on symptoms and neuromuscular control in patients with more severe conditions remain unclear. Third, participants’ expectations of improvement may have affected the actual effects of the training.

## Conclusions

Performing 6 weeks of sling exercise training reduced pain and disability in patients with CLBP and improved the anticipatory activation of the trunk muscles during rapid arm movement. The anticipatory activation improvement was associated with greater functional connectivity between the motor cortex and trunk muscles in patients with CLBP.

## Supporting information

S1 Checklist TREND statement checklist. (PDF)

S1 File (DOC)

S2 File (DOCX)
