# Supplementary material for: Effects of sling exercises on pain, function, and corticomuscular functional connectivity in individuals with chronic low back pain- preliminary study
Source: PLoS One. 2023 Nov 30;18(11):e0288405. doi: 10.1371/journal.pone.0288405 (PMC10688743; doi:10.1371/journal.pone.0288405)
Supplement: S1 File — (DOC) [file pone.0288405.s002.doc]

簽名：

|  | **佛教慈濟醫療財團法人台北慈濟醫院** |
| --- | --- |

人體試驗計畫申請書

（請以中文撰寫，專有名詞儘量附中英對照）

| IRB案號 | | 03-XD14-039 | | | | | | | | | | |
| --- | --- | --- | --- | --- | --- | --- | --- | --- | --- | --- | --- | --- |
| 收件日期 | | _____年____月____日（由人體試委員會填寫） | | | | | | | | | | |
| 計畫  名稱 | 中文 | 懸吊訓練對非特異性慢性下背痛患者的疼痛、功能和神經肌肉控制成效 | | | | | | | | | | |
|  | 英文 | The effect of suspension exercise for non-specific chronic low back pain patient on pain, function and neuromuscular control | | | | | | | | | | |
| 受試者人數 | | 健康成年人15人，有非特異性慢性下背痛患者15人。 | | | | | | | | | | |
| 試 驗 期 間 | | 2014年　7月　1日　至　　2015年　12月　31日 | | | | | | | | | | |
| 計劃主持人 | | 中文姓名：陳柏禎 | | | | | | 英文姓名：Bo-Jhen Chen | | | | |
|  | | 單位：台北慈濟醫院復健科 | | | | | | | | | | |
|  | | 聯絡電話：3516 | | | | | | E-mail：vertigo9371@gmail.com | | | | |
|  | | GCP相關訓練證明　□有 ■無 | | | | | | | | | | |
| 經費贊助者 | | □　無 | | | | | | | | | | |
|  | | ■ 有，名稱：  □藥品/設備製造商:____________________  □衛生署 □國科會 □國家衛生研究院  ■院內 □其他:_______________________ | | | | | | | | | | |
| 研究成員 | | 姓名 | | 單位 | | | 電話/分機 | | | e-mail | | 傳真 |
| 協同主持人 | | 周立偉 | | 國立陽明大學物理治療暨輔助科技學系 | | | 2826-7000 #7092 | | | lwchou@nycu.edu.tw | |  |
| 聯絡人 | | 劉子瑛 | | 國立陽明大學物理治療暨輔助科技學系 | | | 0975816120 | | | v0633@hotmail.com | |  |
| 相關文件最新版本、日期  （若有，請圈選註記） | | ■計畫書 | | ■受試者同意書 | | | □個案報告表 | | | □主持人手冊 | | □廣告文件 |
| **研究背景** | | 有大約8成的人在其一生會發生下背痛，其中8成5的慢性下背痛患者是沒有確切診斷，稱為非特異性慢性下背痛，而慢性下背痛的患者可能會有神經肌肉控制的問題，包含大腦結構與功能改變以及肌肉活性等變化，現今對於改善慢性下背痛患者神經肌肉控制、疼痛、功能的治療方式多以合併治療方式為主，其中合併核心穩定運動有越來越廣泛的趨勢，且其比起只做傳統儀器治療更有效，懸吊訓練是核心穩定訓練的其中一種，然而目前只有少數關於懸吊訓練對於下背痛的研究文章，而且關於為何核心穩定運動對於下背痛的症狀有改善的作用，目前的研究缺乏明確的機制來說明。 | | | | | | | | | | |
| **研究目的** | | 本篇研究的目的為:  1.探討健康成年人與非特異性下背痛患者在神經肌肉控制方面的差異。  2.探討懸吊運動介入對非特異性慢性下背痛患者，在核心肌肉控制、疼痛與功能上成效。 | | | | | | | | | | |
| **研究型態　(多選)** | | - **查驗登記** - **上市後監測**(PMS) - **學術研究** - **有衛生署許可證** - **無衛生署許可證** - 侵入性 - 非侵入性 | | | - 全球多中心 - 國內多中心 - 本院單一中心 - 回溯性 - 延伸試驗 - 前瞻性 - 記錄型 | | | | | - 流行病學 - 問卷型 - 基因相關研究 - 附加試驗 - 研究用人體新採檢體 - 研究用人體剩餘檢體 - 其他：________ | | |
| **試驗品項簡介** | | - 已有衛署許可證字號醫藥或醫材產品，請說明 - 新藥，名稱 - 新醫療器材，名稱 - 新醫療技術，名稱 - 中草藥，名稱 - 食品，名稱 - 其它，請說明 沒有使用試驗品 | | | | | | | | | | |
| **使用幅射物質** | | ■ 無  ⬜ 只限醫療用途 | | | | | | | | | | |
| **本計劃是否(已/將)在其它單位送審** | | ■ 無  ⬜ 有（哪些單位及其審查結果︰ ）  ⬜ 規劃中（預定哪些單位︰ ） | | | | | | | | | | |
| **是否須送衛生署審查** | | - 是   ■ 否 | | | | | | | | | | |
| **研究設計** | | 觀察型 | □ 個案對照研究 | | | | | | □ 世代族群研究 | | | |
|  |  |  | □ 描述性研究（無對照組研究） | | | | | | | |  | |
|  |  | 介入型 | ■ 對照 | | | | □ 非對照 | | | |  | |
|  |  |  | □ 平行 | | | | □ 交叉 | | | |  | |
|  |  |  | □ 開放 | | | | □ 單盲 | | | | □ 雙盲 | |
|  |  |  | ■ 隨機 | | | | □ 非隨機 | | | |  | |
|  |  |  | □ 較優性 | | | | □ 不劣於 | | | | □ 相等性 | |
|  |  |  | - phase Ι - phase Ⅳ | | | | - phase Π - 先導性(pilot) | | | | - phase Ш - 其他： | |
|  |  |  | 是否有Data Safety Monitoring Board | | | | | | | | □ 是 ， ■ 否 | |
|  |  | 其它（請說明）： | | | | | | | | | | |
| **研究程序** | | 第一階段:  招募15位年滿20歲的健康成年人，於受試者簽署受試者同意書後，分別在懸吊運動前、訓練經過4、8周後，以及訓練結束後4周，收取其在執行功能性動作任務、肌力測試時的肌電圖和腦波圖訊號。(功能性動作任務包含站立下快速手臂上舉、重複彎腰上下搬動重物於雙膝和胸口間；肌力測試包含腹、背肌)  第二階段:  招募15位非特異性慢性下背痛患者。  1. 於受試者簽署受試者同意書後，收取其在執行功能性任務、肌力測試時的肌電圖和腦波圖訊號。  2. 在介入組經由懸吊訓練後4、8周，以及懸吊訓練結束後4周，收取其在執行功能性任務時的肌電圖和腦波圖訊號。對照組於第一次收取資料後每4周再次收取資料。  (懸吊訓練的原則是以各個受試者的能力來決定，動作包含單純腹橫肌收縮、平板式、空中腳踏車等等。) | | | | | | | | | | |
| **樣本數量** | | - 全球_______人 | | | - 國內__15__人 | | | | | - 本院__40___人 | | |
| **實驗對象** | | - 正常人 | | | - 病患 | | | | | - 弱勢團體 | | |
| **受試者特徵** | | 年齡範圍 | | 年齡範圍：___20___歲～____50____歲 | | | | | | | | |
|  |  | 孩童 | | ■ 無 | | □ < 1歲 | | | | □ 1-3歲 | | □ 4-14歲 |
|  |  | 殘障 | | ■ 無 | | □ 生理 | | | | □ 認知 | | □ 心理 |
|  |  | 孕婦 | | □ 是 | | ■ 否 | | | |  | |  |
|  |  | 安養院收容人 | | □ 是 | | ■ 否 | | | |  | |  |
|  |  | 受刑人 | | □ 是 | | ■ 否 | | | |  | |  |
| **特殊條件** | | - 加護照顧 - 孩童加護照顧 - 基因治療 - 義肢 | | | - 隔離 - 靜脈注射 - 管制藥品 - 其他 __無__ | | | | | - 手術 - 電腦斷層掃描 - 婦科用品 | | |
| **納入條件** | | 第一階段:   1. 健康成年人 (沒有神經肌肉骨骼等疾病。)   2. 年齡介於20至55歲  第二階段:   1. 非特異性慢性下背痛病人 2. 年齡介於20至55歲 | | | | | | | | | | |
| **排除條件** | | 第一階段:  沒有辦法配合研究者  第二階段:  1. 有脊椎骨骼結構上的異常(例如:椎管狹窄)  2. 有神經功能缺損(例如:神經根病變)  3. 有影像學上的診斷  4. 有其他系統性疾病  5. 沒有辦法配合研究者 | | | | | | | | | | |
| **預期研究結果** | | 1.健康成年人其神經肌肉控制能力較非特異性慢性下背痛患者好。  2.有懸吊訓練的介入組會比對照組在核心肌群控制、疼痛與功能評估上有較多的進步。 | | | | | | | | | | |
| **統計分析方法** | | 目的1:  使用SPSS 21.0版作為統計分析工具，利用描述性統計呈現受試者基本資料，並依造不同的資料類型以獨立t檢定(independent t test)或卡方檢定(X^2^ test)進行健康成年人組和非特異性慢性下背痛組間的數據比較。  目的2:  使用SPSS 21.0版作為統計分析工具，利用描述性統計呈現受試者基本資料，並依造不同的資料類型以獨立t檢定(independent t test)或卡方檢定(X^2^ test)進行介入組和對照組間數據比較。針對連續變相的成效分析，如肌電圖、腦波圖、疼痛量表、日常功能性量表、肌力測試，以雙因子重複測量變異數分析(two-way repeated measures analysis of variable)探討兩組間與不同介入週數的成效差異。 | | | | | | | | | | |
| **執行進度及其監督方法** | | 依排定的懸吊訓練介入時間以及與受試者約各個階段收取資料的時間，逐步完成研究。  目的1完成時間約需3~4個月，目的2完成時間約再7~8個月後。 | | | | | | | | | | |
| **受試資料保密方式：**  **(多選)** | | ■ 以編號識別 ⬜ 英文縮寫名識別 ■ 將資料編碼  ⬜ 所有資料上鎖 ■ 編號或編碼之資料上鎖 ⬜ 其他： _____________________________________  除主持人、監視人及協同主持人外會審視受試者資料的名單：  ____________ ____________ ____________ ___________ | | | | | | | | | | |
| **招募受試者方式** | | ⬜ 不適用  ■ 計劃主持人(含協同主持人)口頭介絡  ⬜ 其它醫師護士(非共/協同主持人)口頭介絡  □ 海報廣告(內容是否已附上：⬜是　⬜否)  ⬜ 網路廣告(內容是否已附上：⬜是　⬜否)  ⬜ 其他： _____________________________________ | | | | | | | | | | |
| **受試者同意書** | | 1. ■是，內容已附上。 2. □否，使用免除受試者同意書。 3. □否，有其他原因，請說明 | | | | | | | | | | |
| **受試者同意書取得程序**  **（如使用免除受試者同意書者，本欄位免填）** | | 1. 由誰向受試者或其法定代理人解釋驗內容並取得同意?   ■ 計畫主持人  ■ 協同研究人員（請註明： 劉子瑛 ）  ⬜ 其他醫師（非共同/協同主持人）（請說明： ）  ⬜ 其他研究參與人員（請說明： ）   1. 取得同意時間?   ⬜篩選前，■篩選後，隨機分派前   1. 在什麼地點解釋試驗內容? 每件約花費多少時間?   ⬜ 診間  ⬜ 病房  ⬜ 檢查室  ■ 其它（請說明）：物理治療室  每件花費時間： 10~15分鐘 。   1. 除了簽署受試者同意書以外，如何確保受試者或其法定代理人對試驗內容了解?   ⬜主持人手冊  ■與受試者及其家人共同討論  ■與受試者及協助說明者共同討論  ⬜另安排時間作追蹤  ⬜其他（請說明）： | | | | | | | | | | |
| **計畫主持人聲明** | | 1.以上資料，已盡力確保內容正確。若有不實或蓄意隱瞞，願負法律上應負之責任。  2.本人負責執行此臨床試驗，已仔細閱讀過計畫書。願依赫爾辛基宣言的精神及國內相關法令的規定，確保試驗對象之生命、健康、個人隱私及尊嚴。  3.本人承諾將依計畫書內容執行，並依國內相關法令的規定通報嚴重不良反應事件，繳交期中及期末報告，提供所需的所有相關資訊給佛教慈濟綜合醫院台北分院人體試驗審查委員會，以作確保受試者權益之審核。  4.若以後有任何計畫內容的修改，除了要立即降低危險性的情況外，在未獲得佛教慈濟綜合醫院台北分院人體試驗審查委員會同意前，絕不會進行修改後的內容。  簽　　　名：_______________  日期：___年___月___日 | | | | | | | | | | |
