# Supplementary material for: Effects of sling exercises on pain, function, and corticomuscular functional connectivity in individuals with chronic low back pain- preliminary study
Source: PLoS One. 2023 Nov 30;18(11):e0288405. doi: 10.1371/journal.pone.0288405 (PMC10688743; doi:10.1371/journal.pone.0288405)
Supplement: S2 File — (DOCX) [file pone.0288405.s003.docx]

Signature:

|  | **Taipei Tzu Chi Hospital** |
| --- | --- |

Application for Human Testing Program

**(Please write in Chinese, and compare proper nouns in Chinese and English as much as possible)**

| IRB case number | | 03-XD14-039 | | | | | | | | |
| --- | --- | --- | --- | --- | --- | --- | --- | --- | --- | --- |
| Receipt date | | ______________ | | | | | | | | |
| plan  name | Chinese | 懸吊訓練對非特異性慢性下背痛患者的疼痛、功能和神經肌肉控制成效 | | | | | | | | |
|  | English | The effect of suspension exercise for non-specific chronic low back pain patient on pain, function and neuromuscular control | | | | | | | | |
| Number of participants | | 15 healthy adults and 15 patients with nonspecific chronic low back pain. | | | | | | | | |
| During the trial | | From July 1st, 2014, to December 31st, 2015 | | | | | | | | |
| Plan Host | | Chinese Name: 陳柏禎 | | | | English name: Bo-Jhen Chen | | | | |
|  | | Unit: Department of Rehabilitation, Taipei Tzu Chi Hospital | | | | | | | | |
|  | | Contact number: 3516 | | | | Email:vertigo9371@gmail.com | | | | |
|  | | GCP related training certificate □Yes ■ None | | | | | | | | |
| Funding sponsors | | □ None | | | | | | | | |
|  | | ■ Yes, agency:  □ Drug/Device Manufacturer: ________  □ Department of Health, □ National Science Association, □National Institutes of Health  ■Taipei Tzu Chi Hospital □ Others: ________ | | | | | | | | |
| Research Members | | name | unit | | | Phone/Extension | | email | | fax |
| Co-PI | | Li-Wei Chou | Department of Physical Therapy and Assistive Technology, National Yang Ming Chiao Tung University | | | 2826-7000 #7092 | | lwchou@nycu.edu.tw | |  |
| Contact person | | Ziying Liu | Department of Physical Therapy and Assistive Technology, National Yang Ming Chiao Tung University | | | 0975816120 | | v0633@hotmail.com | |  |
| The most recent version and date of the relevant documents  (If yes, please circle the note) | | ■Project proposal | ■Subject consent form | | | □ Case report | | □ PI Manual | | □ ad document |
| **Research background** | | About 80% of people will have low back pain in their lifetime, of which 85% of patients with chronic low back pain that are not accurately diagnosed, is defined as non-specific chronic back pain. Patients with non-specific chronic low back pain may have neuromuscular control problems, including changes in brain structure and function and muscle activity etc. Currently, the treatment approaches for improving neuromuscular control, pain, and functionality in patients with chronic lower back pain mainly focus on combination therapy. Among them, combining core stability exercises has become increasingly popular, and it is more effective compared to traditional instrument-based treatments alone. Suspension training is one of the core stability training methods. However, there are only limited research articles on the effects of suspension training on lower back pain. Furthermore, the existing research lacks clear mechanisms to explain why core stability exercises have a positive impact on the symptoms of lower back pain. | | | | | | | | |
| **Purpose of the study** | | The objectives of this study are:  1. To explore the differences in neuromuscular control between healthy adults and patients with non-specific low back pain.  2. To explore the effectiveness of suspension exercise intervention in core muscle control, pain and function in patients with non-specific chronic low back pain. | | | | | | | | |
| **Research type (multiple selection).** | | - **For Examination and registration** - **Post-marketing surveillance** (PMS). - **Academic research** - **Department of Health (DH) license** - **No DH license** - Invasive - Non-invasive | | - Global multi-center - Domestic multi-center - Single center at our hospital - Retrospective - Extended testing - Prospective - -Documentation | | | | - epidemiology - Questionnaire type - Genetically-related research - Additional trials - Research with new human specimens - Research with remaining human specimen - Other:________ | | |
| **Introduction to the test item** | | - Pharmaceutical or medical products already licensed by the Department of Health, please specify - New drug, name:________ - New medical device, name:________ - New medical technology, name:________ - Chinese herbal medicine, name:________ - Food, name:________ - Otherwise, please specify: no test product is used | | | | | | | | |
| **Using radioactive material** | | ■ None  ⬜ For medical use only | | | | | | | | |
| **Whether the current project has (be/will) been submitted for review in other institute** | | ■ None  ⬜ Yes (which units and their review results: )  ⬜ Planning (which units are booked.) | | | | | | | | |
| **Whether it needs to be sent to the Department of Health for review** | | - be   ■ No | | | | | | | | |
| **Study design** | | Observational | □ Case-control studies | | | | □ Cohort Studies | | | |
|  |  |  | □ Descriptive studies (no controlled group studies) | | | | | |  | |
|  |  | Intervention type | ■ Comparison | | | ⬜ non-control | | |  | |
|  |  |  | ⬜ parallel | | | ⬜ Crossover | | |  | |
|  |  |  | ⬜ open | | | ⬜ single-blind | | | ⬜ Double blind | |
|  |  |  | ■ Random | | | ⬜ non-random | | |  | |
|  |  |  | ⬜ Superior | | | ⬜ not inferior | | | ⬜ Equality | |
|  |  |  | - phase I - phase Ⅳ | | | - phase II - Pilot | | | - phase Ш - Other: | |
|  |  |  | Is there a Data Safety Monitoring Board | | | | | | ⬜Yes, ■No | |
|  |  | Other (please specify): | | | | | | | | |
| **Research procedures** | | Stage 1 :  Fifteen healthy adults aged 20 years or older were recruited to collect EMG(Electromyography), EEG(Electroencephalography), functional motor tasks and muscle strength tests before the suspension exercise, 4th and 8th week of training, and 4 weeks after the end of the training. (Functional movement tasks include standing with rapid arm raise, repeatedly bending over and moving heavy objects up and down between knees and chest; Strength test including abdominal and back muscles)  Stage 2 :  Fifteen patients with non-specific chronic low back pain were enrolled.  1. After the subjects sign the consent form, we will record EMG and EEG when performing functional tasks and muscle strength tests.  2. Collect EMG and EEG from the intervention group during functional tasks 4th and 8th weeks of suspension training and 4 weeks after suspension training. The control group participate in data collection every 4 weeks after the first collection.  (The contents of suspension training is determined by the ability of each subject, and the movements include simple transverse abdominis contraction, flat plate, aerial biking, and so on.) | | | | | | | | |
| **Sample size** | | - Global _______ people | | - Domestic __15__ people | | | | - __40___ people in this hospital | | |
| **Experimental subjects** | | - Normal | | - Patient | | | | - Vulnerable groups | | |
| **Subject characteristics** | | Age range | Age range: ___20___ years old ~ ____50____ years old | | | | | | | |
|  |  | children | ■ None | | ⬜ < 1 year | | | ⬜ 1-3 years | | ⬜ 4-14 years old |
|  |  | Disability | ■ None | | ⬜ Physiological | | | ⬜ Cognitive | | ⬜ Psychological |
|  |  | pregnant woman | ⬜ Yes | | ■ No | | |  | |  |
|  |  | Nursing home hospices | ⬜ Yes | | ■ No | | |  | |  |
|  |  | Inmates | ⬜ Yes | | ■ No | | |  | |  |
| **Special conditions** | | - Intensive care - Intensive care for children - gene therapy - prosthesis | | - isolation - intravenous injection - Controlled drugs - Other __None__ | | | | - surgery - CT scan - Gynecological supplies | | |
| **Conditions for inclusion** | | Stage 1 :   1. Healthy adults without neuromusculoskeletal diseases.   2. Between 20 and 55 years old  Stage 2 :   1. Patients with nonspecific chronic low back pain 2. Between 20 and 55 years old | | | | | | | | |
| **Exclusion criteria** | | Stage 1 :  Unable to cooperate with the researchers  Stage 2 :  1. Abnormality in the bone structure of the spine (e.g., spinal stenosis)  2. Neurological deficits (e.g., radiculopathy)  3. Have an imaging diagnosis  4. Have other systemic diseases  5. Unable to cooperate with the researchers | | | | | | | | |
| **Expected study results** | | 1. Healthy adults have better neuromuscular control than patients with non-specific chronic back pain.  2. The interventional group with suspension training will have more progress in core muscle control, pain and function assessment than the control group. | | | | | | | | |
| **Methods of statistical analysis** | | Purpose 1:  SPSS 21.0 will be used as statistical analysis tool. Descriptive statistics to present basic data of subjects, and comparing data between healthy adults and non-specific chronic back pain groups using independent t test or chi-square test (X^2^ test) depending on data types.  Purpose 2:  Using SPSS 21.0 as a statistical analysis tool. Descriptive statistics were used to present basic subject data, and different data types were used as independent t test or chi-square (X^2^ test) to compare data between intervention and control groups. For continuous data in time domain, such as EMG, EEG, pain scale, daily functional scale, and muscle strength test, two-way repeated measures analysis of variable(two way ANONVA) will be used to analyze the difference in effectiveness between two groups and the number of weeks of intervention. | | | | | | | | |
| **Implementation progress and its monitoring methodology** | | The study was progressively completed according to the scheduled suspension training intervention time and the time to receive data at each stage of the subject.  The completion time of Objective 1 is about 3~4 months, and the completion time of Objective 2 is about 7~8 months later. | | | | | | | | |
| **Confidentiality method of research data:**  **(multiple selection).** | | ■ Identification by number ⬜Identification by English abbreviation ■ Data encoding  ⬜All data are locked ■ Numbered or coded data is locked Other: ____⬜____  In addition to moderators, monitors, and co-moderators, a list of subjects whose profiles will be reviewed:  ____________ ____________ ____________ ___________ | | | | | | | | |
| **Subjects recruitment method** | | ⬜ not applicable  ■ Oral introduction by the PI (including co-host).  ⬜ Oral introduction by other physicians and nurses (non-communist/co-moderator)  ⬜ Poster ad (content attached: Yes No)⬜⬜  ⬜ Online advertising (content attached: Yes No)⬜⬜  ⬜ Other: ______ | | | | | | | | |
| **Subject consent form** | | 1. ■Yes, the contents are attached. 2. ⬜No, use the exempt subject consent form. 3. ⬜No, there are other reasons, please specify | | | | | | | | |
| **Procedure for obtaining consent forms**  **(If exempted from the use of consent form, this field is not required)** | | 1. Who explains the test to the subject or his/her legal representative and obtains consent?   ■ PI  ■ Associate researchers (please specify: Ziying Liu ).  ⬜ Other physicians (non-co-PI) (please specify: )  ⬜ Other staff (please specify: )   1. When to get consent?   ⬜ Before screening, ■ After screening, before random assignment   1. Where is the test content interpreted? How much time does it take?   ⬜ Out-patient clinic  ⬜ ward  ⬜ Examination room  ■ Others (please specify): Physical therapy room  Time taken: 10~15 minutes.   1. In addition to signing the subject's consent form, how to ensure that the subject or his/her legal representative is aware of the trial content?   ⬜ Moderator Handbook  ■ Discuss with the subject and his or her family  ■ Discuss with the subject and the interpreter  ⬜ Schedule additional time for tracking  ⬜ Other (please specify): | | | | | | | | |
| **PI Statement** | | 1. Every effort has been made to ensure that the above information is correct. If there is any false or deliberate concealment, it is willing to bear legal responsibility.  2. I am responsible for the execution of this clinical trial and have carefully read the project proposal. In accordance with the Declaration of Helsinki and the provisions of relevant domestic laws and regulations, we are willing to ensure the life, health, personal privacy and dignity of test subjects.  3. I promise to implement the content of the plan, report serious adverse reaction events in accordance with the relevant laws and regulations, submit the interim and final reports, and provide all relevant information to the Human Trial Review Committee of the Taipei Branch of Buddhist Tzu Chi General Hospital to ensure the rights and interests of subjects.  4. If there is any future revision of the planned content, except in cases where the risk is to be immediately reduced, the revised content will not be carried out without the approval of the Human Test Review Committee of the Taipei Branch of Tzu Chi General Hospital of Buddhism.  Signed: ________  Date: ___year___month___day | | | | | | | | |
